# Neurorestoration of Sustained Attention in a Model of HIV-1 Associated Neurocognitive Disorders

**DOI:** 10.3389/fnbeh.2019.00169

**Published:** 2019-08-06

**Authors:** Landhing M. Moran, Kristen A. McLaurin, Rosemarie M. Booze, Charles F. Mactutus

**Affiliations:** Program in Behavioral Neuroscience, Department of Psychology, University of South Carolina, Columbia, SC, United States

**Keywords:** S-Equol, gut-brain axis, phytoestrogen, dose response, HIV-1 transgenic rat

## Abstract

Due to the sustained prevalence of human immunodeficiency virus (HIV)-1 associated neurocognitive disorders (HAND) in the post-combination antiretroviral therapy (cART) era, as well as the increased prevalence of older HIV-1 seropositive individuals, there is a critical need to develop adjunctive therapeutics targeted at preserving and/or restoring neurocognitive function. To address this knowledge gap, the present study examined the utility of S-Equol (SE), a phytoestrogen produced by gut microbiota, as an innovative therapeutic strategy. A signal detection operant task with varying signal durations (1,000, 500, 100 ms) was utilized to assess sustained attention in HIV-1 transgenic (Tg) and control animals. During the signal detection pretest assessment, HIV-1 Tg animals displayed profound deficits in stimulus-response learning and sustained attention relative to control animals. Subsequently, between 6 and 8 months of age, HIV-1 Tg and control animals were treated with a daily oral dose of either placebo or SE (0.05, 0.1, 0.2 mg) and a posttest assessment was conducted in the signal detection operant task with varying signal durations. In HIV-1 Tg animals, a linear decrease in the number of misses at 100 ms was observed as SE dose increased, suggesting a dose response with the most effective dose at 0.2 mg SE, approximating controls. Comparison of the number of misses across signal durations at the pretest and posttest revealed a preservation of neurocognitive function in HIV-1 Tg animals treated with 0.2 mg SE; an effect that was in sharp contrast to the neurocognitive decline observed in HIV-1 Tg animals treated with placebo. The results support the utility of 0.2 mg SE as a potential efficacious neuroprotective and/or neurorestorative therapeutic for sustained attention, in the absence of any adverse peripheral effects, in the HIV-1 Tg rat. Thus, the present study highlights the critical need for further *in vivo* studies to elucidate the full potential and generalizability of phytoestrogen treatment for HAND.

## Introduction

Worldwide, approximately 36.7 million individuals are living with human immunodeficiency virus type 1 (HIV-1; UNAIDS, 2017), including approximately 4.2 million older adults (>50 years of age; UNAIDS, 2014). Combination antiretroviral therapy (cART), introduced in 1996, serves as the primary treatment regimen for HIV-1 seropositive individuals and dramatically shifted the epidemiological features of both HIV-1 (Justice, 2010) and HIV-1 associated neurocognitive disorders (HAND). Specifically, cART increased the life expectancy for HIV-1 seropositive individuals (e.g., Romley et al., 2014; Teeraananchai et al., 2017) and decreased the prevalence of the most severe forms of neurocognitive impairment (NCI; Ances and Ellis, 2007). Milder forms of NCI (i.e., HAND), however, persist (Cysique et al., 2004; Garvey et al., 2009; Heaton et al., 2011), afflicting between 40% and 70% of HIV-1 seropositive individuals (Letendre et al., 2010; McArthur et al., 2010; Heaton et al., 2011). Due to the sustained prevalence of HAND in the post-cART era, as well as the increased prevalence of older HIV-1 seropositive individuals, there is a critical need to develop adjunctive therapeutics targeted at slowing/preventing neurocognitive decline and/or restoring neurocognitive function.

The gut-brain axis (or the gut-brain-microbiota axis) is a complex network that includes the central nervous system (CNS), the enteric nervous system, and the gastrointestinal tract (Mayer et al., 2015). Trillions of gut microbiota, which comprise the gastrointestinal tract, play a prominent role in the gut-brain axis. Functionally, gut microbiota have been implicated in the modulation of neurocognitive functions (e.g., Gareau et al., 2011; Manderino et al., 2017), emotional behavior (e.g., anxiety: Diaz Heijtz et al., 2011; Neufeld et al., 2011; depression: Kelly et al., 2016), and social interactions (e.g., Tung et al., 2015). Furthermore, alterations in microbiome composition have been observed in multiple neurodegenerative diseases (e.g., HIV-1: Gori et al., 2008; Mutlu et al., 2014; Parkinson’s disease: Keshavarzian et al., 2015; Perez-Pardo et al., 2018; Alzheimer’s disease: Harach et al., 2017).

Alterations in the gut microbiome in HIV-1 seropositive individuals, independent of treatment with cART, include decreased diversity (e.g., Mutlu et al., 2014; Monaco et al., 2016; Hamad et al., 2018) and prominent alterations in gut microbiome composition (e.g., Gori et al., 2008; Dillon et al., 2014; Hamad et al., 2018). Gut dysbiosis in HIV-1 has been associated with both CD4+ T-cell count (e.g., Pérez-Santiago et al., 2013; Nowak et al., 2015) and inflammation (e.g., Ancuta et al., 2008; Dinh et al., 2015; Nowak et al., 2015); pathologic indicators correlated with NCI (e.g., Marcotte et al., 2003; Abassi et al., 2017; Eckard et al., 2017). However, antiretroviral therapy seems to at least partially restore gut integrity (Guadalupe et al., 2006). Furthermore, targeting the gut microbiota *via* probiotic supplementation enhanced neurocognitive function during a pilot study in HIV-1 seropositive individuals (Ceccarelli et al., 2017); results which validate the potential utility of targeting the gut-brain axis for the development of adjunctive therapeutic treatments for NCI commonly observed in HIV-1 seropositive individuals.

sDue to the reported gut dysbiosis in HIV-1 seropositive individuals Equol, the active metabolite produced by the gut microbiota following ingestion of the soy-derived phytoestrogen daidzein (Setchell et al., 1984), was assessed as an adjunctive therapeutic approach for HAND. Although Equol can exist in either the R- or S- conformation, given its chiral center at carbon 3, S-Equol (SE) is the only enantiomer produced by humans (Setchell et al., 2005). The neuroprotective effects of SE occur *via* its selective affinity for estrogen receptor β (ERβ), with SE showing an even greater affinity for ERβ than daidzein (Setchell et al., 2005). Functionally, the benefits of a soy food-based diet, such as a reduced risk for certain cancers and increased bone density, are typically found in adults who produce SE (Setchell et al., 2002; Lampe, 2009; Jackson et al., 2011), representing approximately 25%–30% of the Western population (Rowland et al., 2000; Setchell and Cole, 2006). The caveat that therapeutic efficacy and potential may be limited as only 25%–30% of the adults of western countries convert daidzein to SE is fully embraced with our emphasis on use of the metabolite SE, rather than the parent compound, rendering moot the concern of heterogeneity of responses in human gut microbiota as well as any potential differences in gut microflora induced by HIV and/or aging.

Thus, the present study addressed three key questions in the HIV-1 Tg rat. First, do HIV-1 Tg animals exhibit an impairment in sustained attention relative to control animals? Assessments of sustained attention have revealed prominent alterations in both HIV-1 seropositive children (Watkins et al., 2000) and adults (e.g., Fein et al., 1995); deficits which were characterized by a failure of response inhibition (Watkins et al., 2000) and alterations in the temporal dimension of attention (Fein et al., 1995). Furthermore, in the HIV-1 Tg rat, profound alterations in sustained attention (Moran et al., 2014; McLaurin et al., 2017b, 2019a), have been previously reported. Second, utilizing a dose-response experimental design (i.e., placebo, 0.05, 0.1, or 0.2 mg SE), is SE an efficacious therapeutic for the treatment of sustained attention deficits in the HIV-1 Tg rat? *In vitro* studies demonstrate the neuroprotective and/or neurorestorative properties of daidzein (Bertrand et al., 2014) and SE (Bertrand et al., 2015) in rat neuronal cultures treated with the HIV-1 protein, Tat. Doses selected for the present experiment yielded a daily amount of 0.25–1.0 mg/kg SE; an amount equivalent to a 2.5–10 mg dose in a 60 kg human (see most elderly Japanese have a daily isoflavone intake of 30–50 mg, Akaza, 2012). Third, how does the most effective dose of SE change sustained attention across time (i.e., Pre-SE vs. Post-SE) in HIV-1 Tg animals? It was hypothesized that SE may support an efficacious neurorestorative therapeutic for at least a subset of HIV-1 Tg animals.

## Materials and Methods

### Animals

The efficacy of SE as a neuroprotective and/or neurorestorative treatment for NCI was assessed in ovariectomized (OVX) female Fisher (F344/N; Harlan Laboratories Inc., Indianapolis, IN, USA) rats (HIV-1 Tg, *n* = 41; control, *n* = 43). Due to health issues, two HIV-1 Tg animals were euthanized prior to beginning treatment with SE, yielding HIV-1 Tg, *n* = 39 and control, *n* = 43 for the post-SE assessments. The HIV-1 Tg rat, originally reported in 2001 by Reid et al. (2001) expresses seven of the nine HIV-1 genes constitutively throughout development and displays intact functional health through advanced age (i.e., 18 months of age; Peng et al., 2010; McLaurin et al., 2018a, 2019a). Although the deletion of two viral proteins, including -*gag* and –*pol*, renders the HIV-1 Tg rat non-infectious, it resembles HIV-1 seropositive individuals on cART and serves as a valid and reliable animal system to translationally model NCI commonly observed in HAND (e.g., Vigorito et al., 2007; Moran et al., 2013, 2014; Repunte-Canonigo et al., 2014; McLaurin et al., 2017b, 2019a). Animals were received at the animal vivarium at approximately 2-months of age and were pair- or group-housed throughout the duration of sexperimentation.

All rats were OVX at Harlan Laboratories prior to arrival at the animal vivarium. Given that SE is a nonsteroidal estrogen, with preferential binding to ERβ (Setchell and Clerici, 2010), OVX animals were utilized to preclude the potential confounding effect of endogenous hormones. Additionally, HIV-1 Tg and control animals were fed a minimal phytoestrogen diet [≤20 ppm; Teklad 2020X Global Extruded Rodent Diet (Soy Protein-Free)] due to the structural similarities between phytoestrogen and estrogen. Beginning approximately 1 week before preliminary training, animals were placed on food restriction to maintain 85% of their *ad libitum* body weight. Pair- or group housing was maintained during food restriction. Water was available *ad libitum* throughout the duration of the study.

Animals were maintained according to National Institute of Health (NIH) guidelines in AAALAC-accredited facilities. The animal facility was maintained at 21° ± 2°C, 50% ± 10% relative humidity and had a 12-h light:12-h dark cycle with lights on at 07:00 h (EST). The project protocol was approved under federal assurance (#D16-00028) by the Institutional Animal Care and Use Committee (IACUC) at the University of South Carolina.

### Apparatus

Operant training was conducted in 22 operant chambers located inside sound-attenuating chambers (Med Associates Inc., Fairfax, VT, USA). The front wall of each chamber had two retractable levers, a pellet dispenser (45 mg), located between the two levers, and three incandescent panel lights (20 ± 2 lux). The central panel light, located above the pellet dispenser, was used in the present experiment for the presentation of signals. A house light was located at the top of the rear wall of the operant chamber. Signal presentation, lever operation, reinforcement delivery, and data collection were controlled by a PC and Med-PC for Windows software (V 4.1.3; Med Associates, Inc., Fairfax, VT, USA).

### Preliminary Training

#### Shaping

Beginning at approximately 3 months of age, animals were trained to lever-press using a standard shaping response protocol. The house light was illuminated throughout the duration of the 42-min test session. Animals were trained to press both levers on an FR-1 schedule of reinforcement for sucrose pellets (45 mg). To prevent side-bias, animals were limited to no more than five consecutive presses on a single lever. Successful acquisition of shaping required animals to achieve at least 40 reinforcers for three consecutive days, with less than 20% variance across days.

#### Signal Detection Task: Training

A signal detection operant task was utilized to train HIV-1 Tg and control animals on two light stimulus contingencies [i.e., central panel light illumination (signal) vs. no illumination (non-signal)]. A 160 trial test session was conducted in a darkened operant chamber, beginning with a 5 min habituation period. Signal presentation was randomized across trials throughout the session. During the signal trials, the central panel light was illuminated until the animal made a response, or until the levers retracted, whichever occurred first. Two seconds after each trial began, levers were extended until the animal made a response, or 6 s elapsed, whichever occurred first; during which time the light stimulus also remained illuminated during signal trials. Levers were retracted between trials [Intertrial Interval (ITI): 9 ± 3 s]. For half of the animals, responses on the left lever during signal trials (Hits) and on the right lever during non-signal trials (Correct Rejections) were reinforced with a sucrose pellet. In the same manner, responses on the right lever during signal trials (Misses) and on the left lever during non-signal trials (False Alarms) were not reinforced. The reverse set of rules was used for the other half of the subjects.

During training, if an animal responded incorrectly, they were given correction trials, which included up to three repetitions of the trial. If an animal failed to respond appropriately during the correction trials, a force-choice trial occurred. During the forced-choice trial, the same stimulus type was repeated (signal or non-signal) but only the correct lever was extended and remained extended until a correct response was made or 120 s elapsed, whichever occurred first.

HIV-1 Tg and control animals were trained for up to 1 month of daily testing. Animals were required to achieve at least 70% accuracy on three consecutive test sessions to be promoted (adapted from Arnold et al., 2003) to the signal detection task tapping sustained attention. Accuracy was calculated as the total number of hits and correct rejections divided by the total number of responses in a session. Animals had prior experience with basic signal detection, discrimination learning, reversal learning, and extradimensional shift tasks, as previously reported (Moran et al., 2014).

### Experimental Design

**Figure 1:**
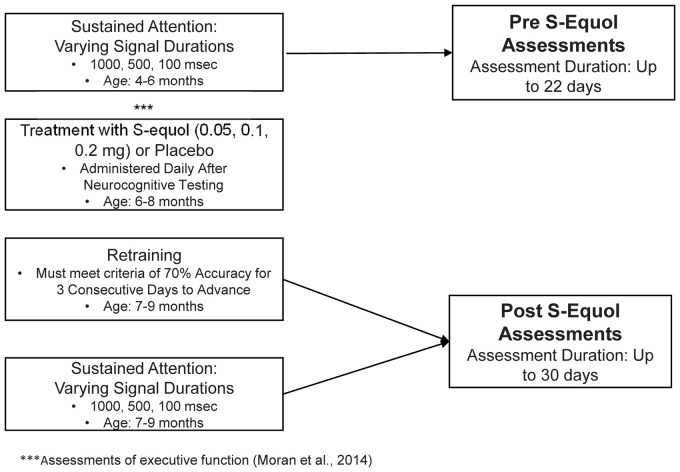
Schematic of the experimental design.

A schematic of the experimental design illustrates the timeline for neurocognitive assessments and treatment with SE, the age of the animals during each phase of the experiment, and the length of each experiment (Figure 1).

### Pre-S-Equol: Sustained Attention Assessments

#### Procedure

After reaching the criterion of 70% accuracy on three consecutive days during preliminary training, animals were assessed in a signal detection operant task with varying signal durations, tapping sustained attention. Each test session was conducted in a darkened operant chamber, beginning with a 5 min habituation period. The length of the light stimulus was manipulated (i.e., 1,000, 500, 100 ms) using a block randomized experimental design across the 162 trial test session. Following signal offset, both levers were presented for 6 s or until the animal made a response, whichever occurred first. Trials had ITIs of 9 ± 3 s, during which time the levers remained retracted. Each animal was trained on the task for upto 22 days.

### Post S-Equol: Sustained Attention Assessments

#### Drugs

SE was obtained from Cayman Chemical Company (Ann Arbor, MI, USA) and incorporated into 90 mg sucrose pellets by Bio-Serv (Flemington, NJ, USA) to produce pellets containing 0.05 mg SE. Plain 90 mg sucrose pellets were also obtained from Bio-Serv for the placebo group.

#### Treatment

Beginning between approximately 6 and 8 months of age, HIV-1 Tg and control animals were treated daily with placebo or SE (0.05, 0.1, or 0.2 mg). A randomized-block experimental design, with percent accuracy on the signal detection operant task with varying signal duration as the blocking factor, was utilized to assign animals to dose groups (i.e., Placebo: Control, *n* = 10, HIV-1 Tg, *n* = 9; 0.05 mg SE: Control, *n* = 11, HIV-1 Tg, *n* = 10; 0.1 mg SE: Control, *n* = 11, HIV-1 Tg, *n* = 10; 0.2 mg SE: Control, *n* = 11, HIV-1 Tg, *n* = 10). Given that each SE pellet contained 0.05 mg, the 0.05 mg dose group received one pellet per day, the 0.1 mg dose group received two pellets per day, and the 0.2 mg dose group received four pellets per day. The placebo group received four sucrose pellets per day. Doses selected for the present experiment yielded a daily amount of 0.25–1.0 mg/kg SE; an amount equivalent to a 2.5–10 mg dose in a 60 kg human (see most elderly Japanese have a daily isoflavone intake of 30–50 mg, Akaza, 2012). Treatments were administered to animals at least 1 h after the completion of neurocognitive testing, and were typically consumed immediately.

#### Procedure

After 5 days of SE or placebo treatment, animals were retrained and retested in the signal detection operant task. Training and signal detection were conducted as described above. As in the assessment prior to SE treatment, animals were required to meet criteria of 70% accuracy for three consecutive days in retraining before advancing to the assessment of sustained attention. Animals were given up to 1 month to reacquire both training and the signal detection operant task.

### Peripheral Effects of S-Equol

Potential peripheral effects of SE were assessed by dissecting the uterine horn from the peritoneal cavity of all rats. The uterine horns were separated from the underlying tissue and the uterine body was excised. Wet weights of the uterine horns were taken immediately following removal. A relative uterine weight was calculated for each animal by dividing the uterine weight (mg) by the body weight (g).

### Statistics

*T*-test, analysis of variance (ANOVA) and regression statistical techniques were utilized for the analysis of all data. SPSS Statistics 25 (IBM Corp., Somers, NY, USA) was used for *t*-test and ANOVA statistical analyses. Figures and regression analyses were completed using GraphPad Prism 5 (GraphPad Software, Inc., La Jolla, CA, USA). *R*^2^ values for all linear regression analyses reflect fits to the mean values and weighting with 1/SD^2^. Statistical tests were evaluated against a *p* ≤ 0.05 alpha criterion. Sample sizes were chosen with the goal of sufficient statistical power (>0.80) to maximize the likelihood of detecting neurocognitive alterations resulting from HIV-1 transgene expression. Partial eta squared ($\eta_{\text{p}}^{2}$) is presented as a measurement of effect size.

A series of three analyses were conducted at the pre-SE assessment to evaluate NCI in the HIV-1 Tg rat. First, the temporal process of acquisition, an assessment of stimulus-response learning, was examined using regression analyses. Second, percent accuracy was analyzed using independent samples *t*-tests. Third, the number of responses for each response type (i.e., Hits, False Alarms, Correct Rejections, and Misses) was examined by calculating the average number of responses (and 95% confidence intervals) exhibited, independent of response type; a value reflecting the inability to distinguish response choices. The number of responses exhibited for each response type independently were then compared to the 95% confidence interval.

Sustained attention was assessed by conducting a mixed-factor ANOVA on the signal detection task with varying signal durations, with genotype (i.e., HIV-1 Tg vs. control) and SE dose (i.e., placebo, 0.05, 0.1, 0.2 mg) as between-subjects factors, as appropriate. Response type (i.e., hits and misses), and signal duration (i.e., 1,000, 500, or 100 ms) were included as within-subjects factors, as appropriate. At the pre-SE assessment, analyses were conducted on the number of hits and misses averaged across test sessions seven through nine; a point reflecting when approximately 50% of the control animals achieved criteria in the signal detection task with varying signal durations. At the post-SE assessment, analyses were conducted on an animal’s final 3 days of testing; a point reflecting the maximal effect of SE. Due to *a priori* hypotheses, analyses at the post-SE assessment compared all of the control animals (reflecting the population sampled), independent of SE dose, to the top 40% performing HIV-1 Tg animals at each dose of SE. The HIV-1 Tg animals were chosen by selecting the animals with the highest average percent accuracy on the final three sessions in the signal detection operant task. The Greenhouse-Geisser *df* correction factor (Greenhouse and Geisser, 1959) and orthogonal decompositions were utilized to preclude potential violations of compound symmetry of the repeated-measures factors. Linear regression analyses were conducted to examine the number of misses as a function of signal duration, as well as to examine the effect of genotype and SE dose on the number of misses at 100 ms.

Based on results from our dose response analysis, time response analyses were conducted. Specifically, regression analyses were utilized to compare pre-SE performance to post-SE performance within groups. The performance at these two time points (i.e., pre-SE vs. post-SE) was compared in four groups, including control animals treated with placebo, control animals treated with 0.2 mg SE, HIV-1 Tg animals treated with placebo, and HIV-1 Tg animals treated with 0.2 mg SE.

The peripheral effects of SE were assessed with relative uterine weight, which was calculated by dividing uterine weight (g) by body weight (g). Statistical analyses were conducted on relative uterine weight using a two-way ANOVA with genotype and SE dose as between-subjects factors.

## Results

### Pre S-Equol: Sustained Attention Assessments

#### Presence of the HIV-1 Transgene Significantly Influenced the Temporal Process of Acquisition During the Pre S-Equol Assessment of the Signal Detection Operant Task With Varying Signal Durations

**Figure 2:**
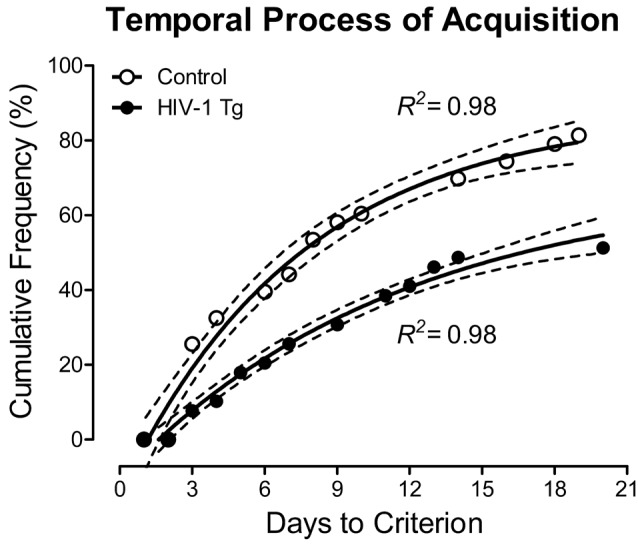
The number of sessions required to meet criterion (70% accuracy on three consecutive sessions) is presented as a function of genotype human immunodeficiency virus (HIV-1 Tg vs. control; ±95% CI). HIV-1 Tg animals exhibited a slower rate of task acquisition relative to control animals, supporting a deficit in stimulus-response learning.

HIV-1 Tg and control animals had up to 22 days to acquire the signal detection operant task with varying signal durations. Across the period of training in the signal detection operant task with varying signal durations, HIV-1 Tg animals exhibited a significantly slower rate of task acquisition, defined as achieving 70% accuracy on three consecutive days, relative to the control group. As illustrated in Figure 2, approximately 55% of the HIV-1 Tg animals acquired the task within the testing period (i.e., 22 days allotted for task acquisition), compared to 81% of the control animals. A one-phase association provided a well-described fit for both HIV-1 Tg (*R*^2^ = 0.98) and control (*R*^2^ = 0.98) animals. However, significant differences in the parameters of the function were observed (*F*_(3,22)_ = 116.1, *p* ≤ 0.001), supporting a profound alteration in stimulus-response learning in HIV-1 Tg animals relative to controls.

#### HIV-1 Tg Animals Exhibited a Marked Impairment in the Detection of Shorter Signal Durations, Supporting a Deficit in Sustained Attention

**Figure 3:**
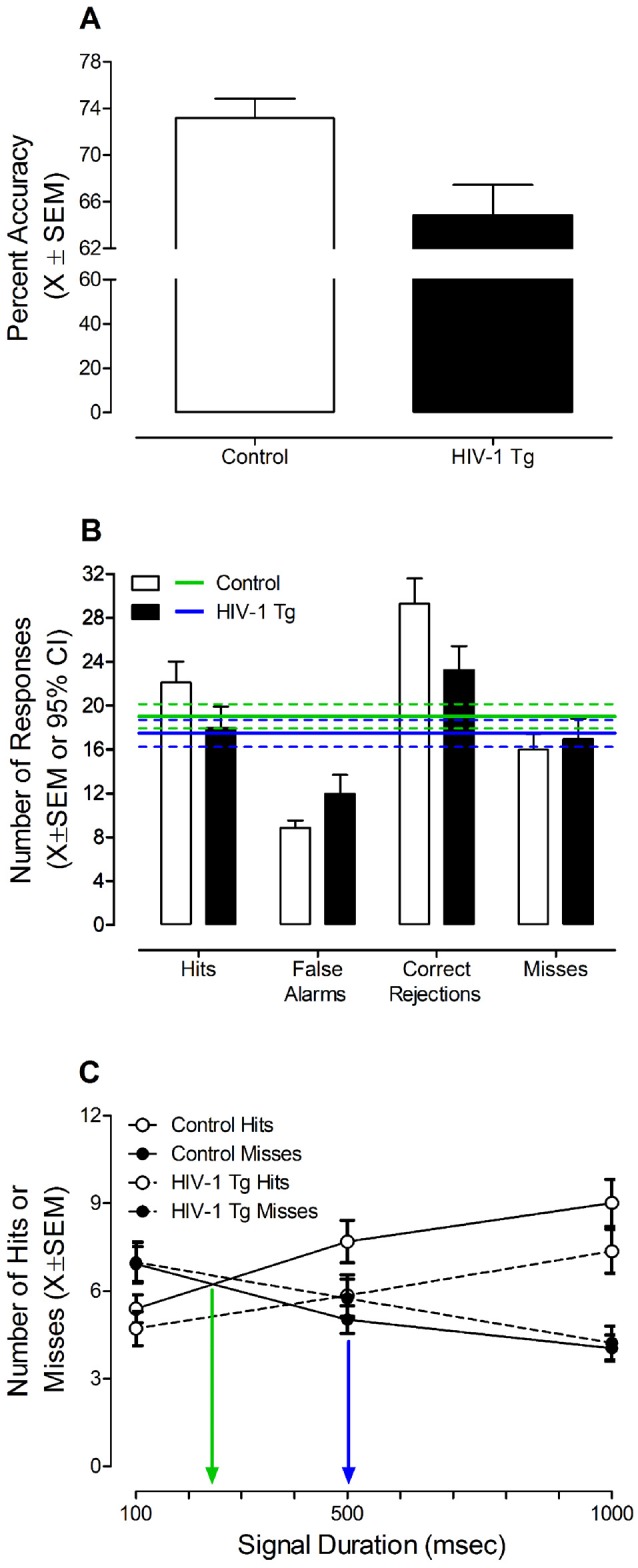
HIV-1 Tg and control animals were tested in a signal detection operant task with varying signal durations prior to treatment with S-Equol. **(A)** HIV-1 animals exhibited a significantly lower percent accuracy relative to control animals (*p* ≤ 0.05). **(B)** Control animals exhibited a significantly greater number of hits and correct rejections, in combination with a significantly fewer number of false alarms and misses, supporting learning all of the task contingencies. HIV-1 Tg animals, however, only exhibited an increased number of correct rejections and a decreased number of false alarms. **(C)** The assessment of sustained attention revealed that HIV-1 Tg animals exhibited a prominent rightward shift in the loss of signal detection (i.e., the intersection of hits and misses; *p* ≤ 0.05) relative to control animals. Data are presented as mean ± SEM.

The effect of the HIV-1 transgene on sustained attention was examined by averaging each animal’s performance from days 7 to 9 on the signal detection operant task with varying signal durations, a point reflecting when half of the control animals met criteria (i.e., 70% accuracy for three consecutive sessions; Figure 3).

Overall, HIV-1 Tg animals (*n* = 33) achieved a significantly lower percent accuracy (Figure 3A) relative to control animals (*n* = 41; *t*_(72)_ = 2.8, *p* ≤ 0.007). Examination of the number of responses for each response type (i.e., Hits, False Alarms, Correct Rejections, and Misses) suggests that HIV-1 Tg and control animals are using different mechanisms to acquire the task (Figure 3B). The number of responses was compared to the average number of responses, independent of response type, and 95% confidence intervals. Control animals exhibited a significantly greater number of hits and correct rejections, in combination with a significantly fewer number of false alarms and misses, supporting learning all of the task contingencies. HIV-1 Tg animals, however, only exhibited an increased number of correct rejections and a decreased number of false alarms.

Presence of the HIV-1 transgene significantly influenced sustained attention during initial training on the signal detection operant task with varying signal durations (Figure 3C). A significant Response Type × Duration interaction (*F*_(2,144)_ = 61.4, *p*_GG_ ≤ 0.001, $\eta_{\text{p}}^{2}$ = 0.460) with a prominent linear component (*F*_(1,72)_ = 78.8, *p* ≤ 0.001, $\eta_{\text{p}}^{2}$ = 0.522), independent of genotype, supports the assessment of sustained attention. As the length of the signal duration decreased, the number of hits decreased and the number of misses increased. Most critically, however, HIV-1 Tg animals exhibited a prominent rightward shift in the loss of signal detection (i.e., where the number of hits and misses intersect) relative to control animals [approximately 479 ms vs. 247 ms, respectively; Genotype × Duration × Response Type Interaction with a prominent quadratic-linear component (*F*_(1,72)_ = 4.3, *p* ≤ 0.05, $\eta_{\text{p}}^{2}$ = 0.056)], supporting a deficit in sustained attention.

During the initial acquisition of the signal detection operant task with varying signal durations, HIV-1 Tg animals exhibited pronounced deficits, characterized by a slower rate of task acquisition, decreased percent accuracy, inability to distinguish hits and misses, and a prominent rightward shift in the loss of signal detection relative to control animals; deficits which suggest profound impairments in stimulus-response learning and sustained attention.

### Post S-Equol: Dose Response

#### In HIV-1 Tg Animals, a Linear Dose Response Was Observed, With the Most Efficacious Dose as 0.2 mg S-Equol

Using a randomized-block design, with percent accuracy on the signal detection operant task with varying signal durations as the blocking factor, HIV-1 Tg and control animals were assigned to one of four SE dose groups (i.e., placebo, 0.05, 0.1, or 0.2 mg SE). After 5 days of treatment, animals were retrained and retested in the signal detection operant task with varying signal durations. Daily SE treatment continued throughout the duration of neurocognitive testing with administration occurring at least 1 h after the completion of assessments.

Given our *a priori* hypothesis that treatment with SE would only mitigate sustained attention deficits in a subset of HIV-1 Tg animals, statistical analyses were conducted on the top 40% of HIV-1 Tg animals at each SE dose. Performance was determined by examining percent accuracy on the final three sessions of SE treatment. All control animals were included in the statistical analysis.

**Figure 4:**
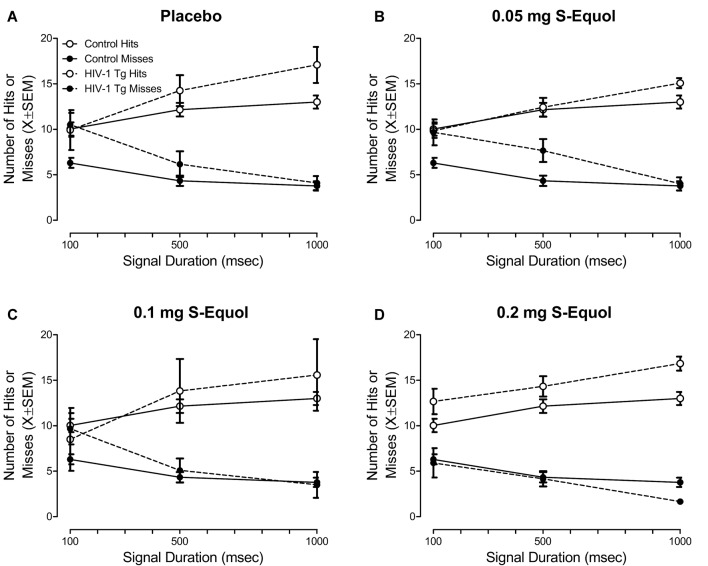
The effect of S-Equol (SE) dose [Placebo **(A)**, 0.05 mg **(B)**, 0.1 mg **(C)**, 0.2 mg **(D)**] on sustained attention was assessed in a signal detection operant task with varying signal durations. There was no significant effect of SE dose on sustained attention in control animals, thus data for control animals were collapsed across all doses. The top 40% of HIV-1 Tg animals, determined using percent accuracy, are presented for each dose. HIV-1 Tg animals continued to exhibit deficits in sustained attention relative to control animals when treated with placebo, 0.05 mg SE, or 0.1 mg SE. However, sustained attention deficits were ameliorated, to the level of controls, in a subset (i.e., 40%) of HIV-1 Tg animals treated with 0.2 mg SE.

Treatment with SE significantly influenced sustained attention, assessed in a signal detection operant task with varying signal durations, in a subset of HIV-1 Tg animals (Figure 4). Specifically, the overall mixed-design ANOVA revealed a significant Genotype × SE Dose × Response Type × Duration interaction with a prominent linear-quadratic component (*F*_(3,48)_ = 4.1, *p* ≤ 0.011, $\eta_{\text{p}}^{2}$ = 0.205).

Complementary analyses were conducted at each SE dose to more fully elucidate the locus of the interaction. A significant Genotype × Duration × Response Type interaction was observed in animals treated with placebo (Figure 4A; *F*_(2,20)_ = 7.7, *p*_GG_ ≤ 0.009, $\eta_{\text{p}}^{2}$ = 0.436) with a prominent linear-linear component (*F*_(1,10)_ = 9.4, *p* ≤ 0.012, $\eta_{\text{p}}^{2}$ = 0.486), 0.05 mg SE (Figure 4B; *F*_(2,26)_ = 4.8, *p*_GG_ ≤ 0.02, $\eta_{\text{p}}^{2}$ = 0.272) with a prominent linear-linear component (*F*_(1,13)_ = 5.8, *p* ≤ 0.032, $\eta_{\text{p}}^{2}$ = 0.309), and 0.1 mg SE (Figure 4C; *F*_(2,26)_ = 9.0, *p*_GG_ ≤ 0.003, $\eta_{\text{p}}^{2}$ = 0.408) with a prominent linear-linear component (*F*_(1,13)_ = 9.5, *p* ≤ 0.009, $\eta_{\text{p}}^{2}$ = 0.421), suggesting that, despite treatment, HIV-1 Tg animals continued to exhibit deficits in sustained attention relative to control animals. The absence of a significant Genotype × Duration × Response Type interaction in animals treated with 0.2 mg SE (Figure 4D; *p* > 0.05) suggested that 0.2 mg SE is the most efficacious dose, ameliorating sustained attention deficits in a subset of HIV-1 Tg animals to the level of controls.

**Figure 5:**
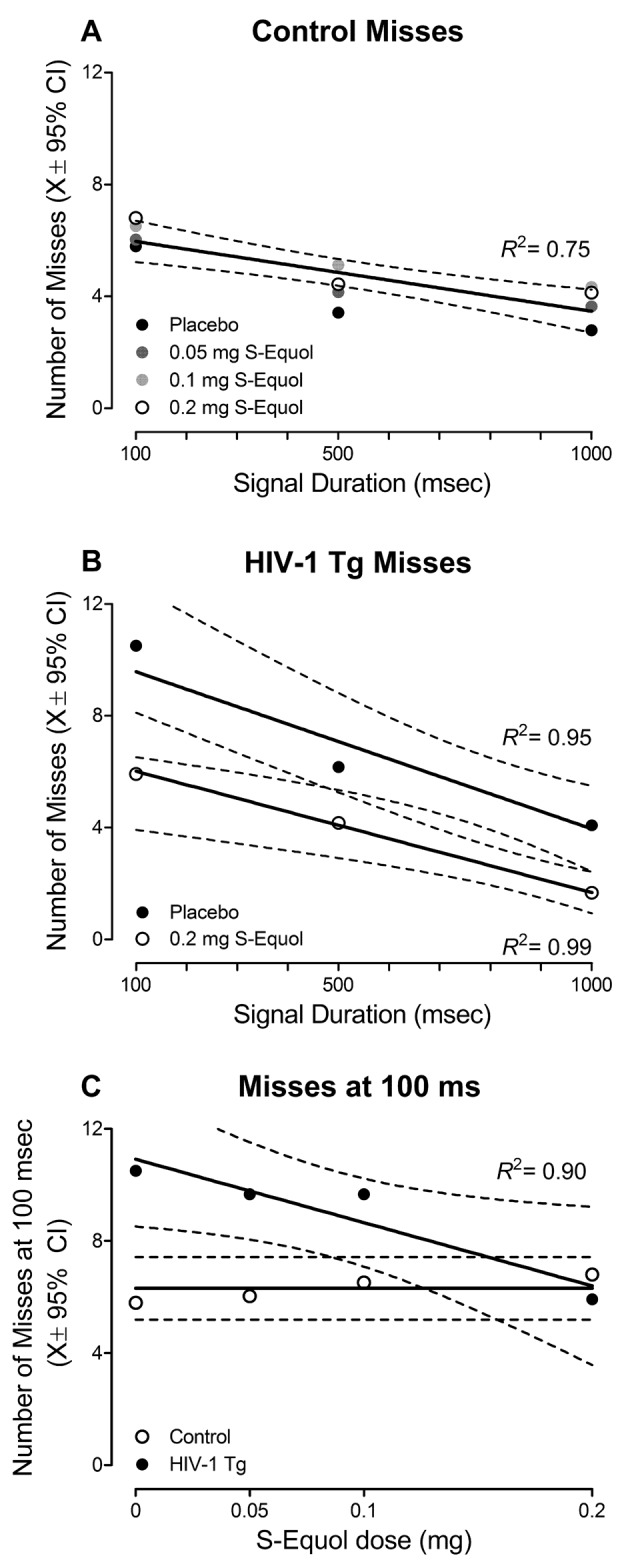
The effect of S-Equol (SE) dose (Placebo, 0.05 mg, 0.1 mg, 0.2 mg) on the number of misses, which reflect a lapse of attention to the stimulus, was examined. **(A)** Treatment with SE did not significantly alter the number of misses across signal duration (i.e., 1,000, 500, 100 ms) in control animals. **(B)** For clarity, only the placebo and 0.2 mg SE dose groups are presented. A downward shift in the number of misses was observed as SE dose increased, with the most prominent shift observed in HIV-1 Tg animals treated with 0.2 mg SE. **(C)** HIV-1 Tg animals displayed a linear decrease in the number of misses at the 100 ms signal duration as the dose of SE increased. A horizontal line, however, provided a well-described fit for the control animals, supporting no change in the number of misses at 100 ms as a function of SE treatment. The number of misses in HIV-1 Tg animals treated with 0.2 mg SE approached the level of controls.

More specific investigations were targeted at examining if, and how, treatment with SE altered the number of misses (Figure 5), given that misses reflect a lapse of attention to the stimulus. A significant Genotype × SE Dose × Duration interaction was observed for misses with a prominent quadratic component (*F*_(3,48)_ = 3.3, *p* ≤ 0.027, $\eta_{\text{p}}^{2}$ = 0.172). In control animals (Figure 5A), a global first-order polynomial provided a well-described fit (*R*^2^ = 0.75), supporting no statistically significant differences in the number of misses as a function of SE dose (*p* > 0.05). However, in HIV-1 Tg animals (Figure 5B), a downward shift in the number of misses was observed as SE dose increased. Although a first-order polynomial provided a well-described fit for all SE doses (i.e., Placebo: *R*^2^ = 0.95, 0.05 mg SE: *R*^2^ = 0.99, 0.1 mg SE: *R*^2^ = 0.86, 0.2 mg SE: *R*^2^ = 0.99), significant differences were observed in the parameters of the function (*F*_(6,40)_ = 2.5, *p* ≤ 0.04).

The most prominent difference in the number of misses was observed at the 100 ms duration; an effect that was subsequently investigated (Figure 5C). A horizontal line provided a well-described fit for the control animals, exhibiting a slope (i.e., β_1_) that was not significantly different from 0 (*p* > 0.05). Results in control animals, therefore, support no significant difference in the number of misses at 100 ms as a function of SE dose. In sharp contrast, for HIV-1 Tg animals, as SE dose increased, a linear (*R*^2^ = 0.90) decrease in the number of misses at 100 ms was observed, suggesting a dose response with the most effective dose as 0.2 mg SE to approximate levels of controls.

Treatment with 0.2 mg SE between 6 and 8 months of age, therefore, enhanced sustained attention, to the level of controls, in the top-performing 40% of the HIV-1 Tg animals sampled. Observations of enhanced sustained attention in HIV-1 Tg animals treated with 0.2 mg SE were due, at least in part, to a decreased number of misses, supporting fewer lapses of attention.

### Pre S-Equol vs. Post S-Equol: Time Response

#### HIV-1 Tg Animals Treated With 0.2 mg S-Equol Exhibited a “Savings” of Sustained Attention

**Figure 6:**
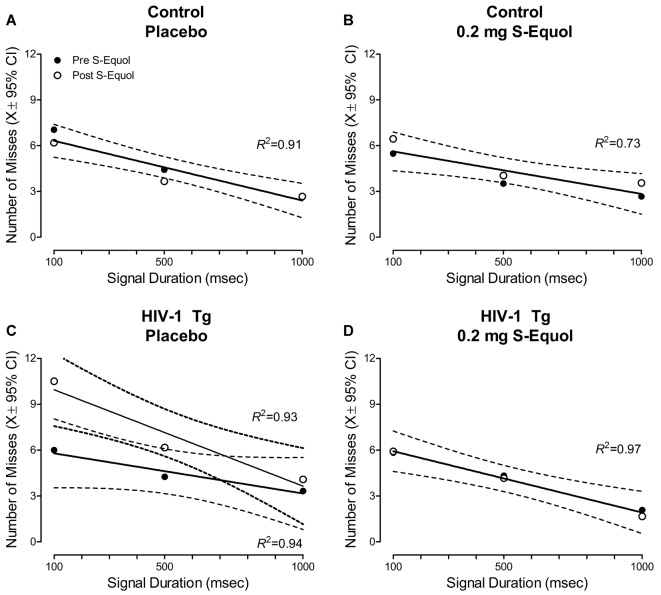
A pretest-posttest experimental design was utilized to examine the effect of S-Equol [SE; Placebo **(A,C)** or 0.2 mg **(B,D)**] on the number of misses across time in control **(A,B)** and HIV-1 Tg **(C,D)** animals. Neither placebo nor 0.2 mg SE treatment altered the number of misses in control animals across time **(A,B)**, evidenced by a global first-order polynomial fit. HIV-1 Tg animals treated with placebo displayed an increase in the number of misses at the post-SE assessment relative to the pre-SE assessment **(C)**, supporting neurocognitive decline across time; an effect that was prevented by treatment with 0.2 mg SE **(D)**.

Given the beneficial effects of 0.2 mg SE observed in a subset of HIV-1 Tg animals, subsequent analyses compared the number of misses at the pre-SE assessment to the number of misses at the post-SE assessment across signal duration (Figure 6).

In control animals, a global first-order polynomial provided a well-described fit for animals treated with placebo (*R*^2^ = 0.80, Figure 6A) and animals treated with 0.2 mg SE (*R*^2^ = 0.73, Figure 6B). A global fit suggests no significant change in the number of misses as a function of time.

In sharp contrast, HIV-1 Tg animals treated with placebo displayed age-related disease progression, evidenced by an increased number of misses at the post-SE assessment relative to the pre-SE assessment. A first-order polynomial provided a well-described fit for HIV-1 Tg animals treated with placebo at both the pre-SE (*R*^2^ = 0.94) and post-SE (*R*^2^ = 0.93) assessment, however, significant differences in the parameters of the function were observed (*F*_(2,20)_ = 4.4, *p* ≤ 0.026). Treatment with 0.2 mg of SE between 6 and 8 months of age, however, prevented neurocognitive decline, evidenced by a global first-order polynomial fit (*R*^2^ = 0.97). Thus, HIV-1 Tg animals treated with 0.2 mg of SE exhibited a “savings” of sustained attention.

### Peripheral Effects of S-Equol: Uterine Weight

#### S-Equol Had no Peripheral Effects, Assessed Using Uterine Weight, in Either HIV-1 Tg or Control Animals, Independent of Dose

**Figure 7:**
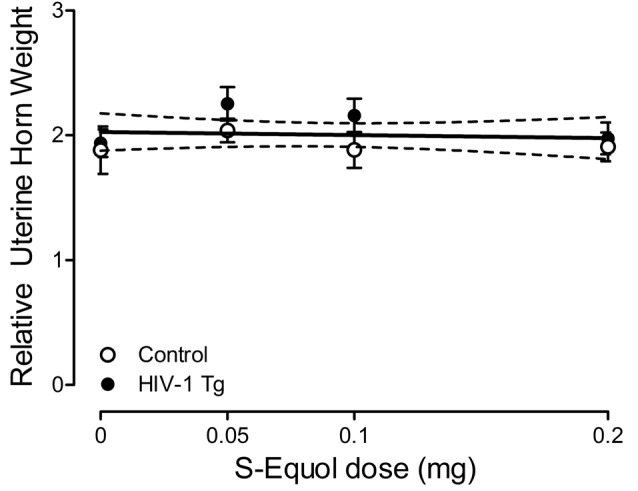
Peripheral effects of S-Equol (SE) were examined by assessing the relative uterine horn weight [i.e., uterine weight (g)/by body weight (g)]. There was no significant effect of genotype (*p* > 0.05) or SE dose (*p* > 0.05) on relative uterine weight, suggesting that SE most likely exerts its effects centrally.

Uterine weight was assessed to examine the potential peripheral effects of SE treatment. A relative uterine weight was calculated by dividing uterine weight (g) by body weight (g). A horizontal line provided a well-described fit for relative uterine weight, exhibiting a slope (i.e., β_1_) that was not significantly different from 0 (Figure 7; *p* > 0.05). Furthermore, a between-subject’s ANOVA failed to reveal a significant Genotype × SE Dose interaction (*p* > 0.05) or main effects of either Genotype or SE dose (*p* > 0.05). Thus, independent of dose, SE had no adverse peripheral effects, assessed using relative uterine weight, in either HIV-1 Tg or control animals, suggesting that the treatment is more likely to act centrally.

## Discussion

At the pre-SE assessment, HIV-1 Tg animals exhibited profound deficits in task acquisition, tapping stimulus-response learning, and signal detection with varying signal durations, tapping sustained attention, relative to control animals, replicating and extending our previous reports (Moran et al., 2014; McLaurin et al., 2017b, 2019a). Between 6 and 8 months of age, HIV-1 Tg and control animals were treated with a daily oral dose of SE (placebo, 0.05, 0.1, 0.2 mg SE) and retested in the signal detection operant task with varying signal durations. In HIV-1 Tg animals, a linear decrease in the number of misses at 100 ms was observed as SE dose increased, suggesting a dose response with the most effective dose as 0.2 mg SE to approximate controls. Specifically, treatment with 0.2 mg SE enhanced sustained attention, to the level of controls, in a subset (i.e., 40%) of HIV-1 Tg animals; an improvement that was due, at least in part, to a downward shift in the number of misses. Comparison of the number of misses during the pre-SE and post-SE assessments revealed a preservation of neurocognitive function in HIV-1 Tg animals treated with 0.2 mg SE; an effect that was in sharp contrast to the neurocognitive decline observed in HIV-1 Tg animals treated with placebo. Treatment with 0.2 mg of SE in adulthood, therefore, may serve as an efficacious therapeutic strategy, slowing/preventing deficits in sustained attention and restoring sustained attention to the level of controls in a subset of HIV-1 Tg animals.

Sustained attention, or vigilance, an integral component of cognitive capacity, is the process by which one detects rare, unpredictable, and weak stimuli over long periods of time (Sarter et al., 2001). Changes in performance during a sustained attention task are often related to varying task parameters, including stimulus duration, intensity and frequency; changes which are analogous in both humans and rats (Parasuraman and Davies, 1977; McGaughy and Sarter, 1995; Bushnell, 1998). The experimental paradigm utilized in the present study, which has been validated for the assessment of sustained attention in animals (McGaughy and Sarter, 1995), required animals to attend to a randomly presented stimulus (i.e., central panel illumination), the presence or absence of which indicated the response required (i.e., which lever to press) to receive a reinforcer (i.e., sucrose pellet). Manipulation of the duration of the randomly presented stimulus (i.e., 1,000, 500, 100 ms), a key component in the assessment of sustained attention, afforded a critical opportunity to examine the temporal aspects of attention.

During the pretest assessment, the population of HIV-1 Tg animals sampled exhibited prominent alterations in stimulus-response learning and sustained attention relative to control animals; impairments which model those commonly observed in HIV-1 seropositive individuals (e.g., Fein et al., 1995; Watkins et al., 2000; Heaton et al., 2011). Deficits in stimulus-response learning, observed in HIV-1 Tg animals relative to controls, were characterized by a slower rate of task acquisition, with only 55% of the HIV-1 Tg animals acquiring the task within 22 days. Furthermore, the HIV-1 Tg rat displayed a profound deficit in sustained attention, characterized by a significantly lower percent accuracy, as well as a rightward shift in the loss of signal detection; alterations which extend those previously reported in the HIV-1 Tg rat (Moran et al., 2014; McLaurin et al., 2017b, 2019a).

Temporal processing deficits, assessed using prepulse inhibition (PPI) and gap prepulse inhibition (gap-PPI), have been implicated as a fundamental impairment in HAND (e.g., Chao et al., 2004; Matas et al., 2010; Moran et al., 2013). In PPI and gap-PPI, the manipulation of interstimulus interval (i.e., the time interval between the prepulse and startle stimulus) is utilized to evaluate the construct of temporal processing (McLaurin et al., 2019b). The generality (McLaurin et al., 2017a), progression (Moran et al., 2013; McLaurin et al., 2016, 2018a), and relative permanence (McLaurin et al., 2017a,c) of temporal processing deficits, assessed using PPI and gap-PPI, have been previously reported in the HIV-1 Tg rat across the functional lifespan. Observed alterations in the temporal aspects of attention in the HIV-1 Tg rat, as in the present study, build upon those previously reported in PPI and gap-PPI, establishing a critical need to characterize temporal processing alterations in other executive functions.

Between 6 and 8 months of age, HIV-1 Tg and control animals were treated with a daily oral dose of SE, an active metabolite produced by gut microbiota, using a dose-response experimental design to determine the lowest efficacious dose of SE. Sustained attention deficits were best ameliorated in a subset of HIV-1 Tg animals (i.e., 40%) following treatment with 0.2 mg SE. Specifically, in HIV-1 Tg animals, a downward shift in the number of misses at all signal durations (i.e., 1,000, 500, 100 ms) was observed as SE dose increased; an effect that was most prominent at the 100 ms signal duration. Subsequent time response analyses were conducted by comparing the number of misses at the pre-SE and post-SE assessment. HIV-1 Tg animals treated with 0.2 mg SE exhibited a preservation of neurocognitive function across time; an effect that was in sharp contrast to the neurocognitive decline observed in HIV-1 Tg animals treated with placebo. Critically, SE had neither a beneficial nor an adverse effect on sustained attention in control animals at any dose. Thus, in HIV-1 Tg animals, 0.2 mg SE served as an efficacious therapeutic for the restoration of neurocognitive function and prevention of neurocognitive decline.

The clinical importance of assessing selective estrogen receptor β agonists (SERBAs), including SE, as a therapeutic for HAND cannot be understated. First, selectively targeting ERβ for therapeutics minimizes the risk for undesirable side effects mediated by ERα in reproductive tissues (Schwen et al., 2012). Furthermore, within the brain, SE crosses the blood-brain-barrier and distributes most significantly to the prefrontal cortex in rats (Lund et al., 2001). Second, the therapeutic potential of SERBAs for a wide range of neurocognitive disorders, including Alzheimer’s disease (e.g., George et al., 2013; Zhao et al., 2013), and Parkinson’s disease (e.g., McFarland et al., 2013) has been examined using preclinical animal model systems. Most critically, the translational relevance of SERBAs in the CNS is demonstrated *via* their progression into clinical trials for other neurocognitive disorders, ranging from Alzheimer’s disease (Ausio Pharmaceuticls; NCT03101085) and memory loss (National Institutes on Aging; NCT01723917) to schizophrenia (Eli Lilly; NCT01874756). Given the translational value of preclinical studies on SERBAs, it seems appropriate to evaluate the therapeutic potential of SE for HAND, another neurodegenerative disease (McLaurin et al., 2019a).

The pathogenesis of HAND in the post-cART era is multidimensional, and may include neurotransmitter alterations (e.g., clinical: Kumar et al., 2011; HIV-1 Tg rat: Javadi-Paydar et al., 2017; Sinharay et al., 2017), synaptic dysfunction (e.g., clinical: Gelman et al., 2012; Desplats et al., 2013; HIV-1 Tg rat: Roscoe et al., 2014; McLaurin et al., 2018b, 2019a), and neuroinflammation (e.g., clinical: Meier et al., 2009; Royal et al., 2016; HIV-1 Tg rat: Royal et al., 2012). Functional alterations in neurons, however, more likely underlie the pathophysiology of HAND (Saylor et al., 2016). Pyramidal neurons, which are abundant throughout the prefrontal cortex (Spruston, 2008), a brain region critically involved in attention (Kim et al., 2016), have been examined in the HIV-1 Tg rat. Specifically, synaptic dysfunction in pyramidal neurons in the HIV-1 Tg rat is characterized by decreased branching complexity and profound alterations in synaptic connectivity; alterations which explain significant genotypic variance (McLaurin et al., 2019a).

Mechanistically, SE may exert therapeutic effects by slowing/preventing and/or restoring synaptic dysfunction. Specifically, *in vitro* studies revealed that pretreatment with SE prevented synaptodendritic damage induced by the HIV-1 viral protein, Tat (Bertrand et al., 2015). Other phytoestrogens, including daidzein and liquiritigenin, precursors to SE, also reduced HIV-1 viral protein (i.e., Tat) induced synaptodendritic damage and restored neuronal complexity (Bertrand et al., 2014). More broadly, strong evidence supports the effect of estrogen, including 17β-estradiol, on neuronal network complexity. In the prefrontal cortex, multiple studies reveal that treatment with 17β-estradiol increases dendritic spine density (Hao et al., 2006; Khan et al., 2013; Tuscher et al., 2016), enhances the formation of excitatory glutamatergic synapses (Khan et al., 2013), and induces morphological changes in dendritic spines (Hao et al., 2006). Furthermore, ERβ, the putative mechanism by which SE exerts effects (Setchell et al., 2005; Bertrand et al., 2015), plays a critical role in the modulation of 17β-estradiol on neuronal network complexity (Wang et al., 2018). There remains, however, a critical need for investigating the precise neural mechanism by which SE exerts neurorestorative effects in the HIV-1 Tg rat.

Despite the aforementioned strengths of the present study, a few caveats must be acknowledged. Specifically, the therapeutic efficacy of SE was assessed exclusively in one neurocognitive domain (i.e., sustained attention). HAND, however, is characterized by prominent alterations in multiple neurocognitive domains (e.g., Cysique et al., 2004; Heaton et al., 2011). Although the therapeutic efficacy of SE cannot be generalized across neurocognitive domains within the present study, results highlight the critical need to conduct further studies investigating the utility of SE. Furthermore, assessments were conducted in OVX female HIV-1 Tg and control animals in adulthood. Given prominent sex differences in HAND (e.g., Hestad et al., 2012; Rowson et al., 2016; Royal et al., 2016; McLaurin et al., 2017b; Maki et al., 2018), the ideal therapeutic would effectively ameliorate NCIs in both males and females. However, OVX female HIV-1 Tg and control animals were used to preclude the potential confounding effect of endogenous hormones. Thus, although the present study provides proof-of-concept that SE effectively ameliorates sustained attention deficits in HIV-1 Tg animals, further *in vivo* studies are required to elucidate the full potential of SE for HAND.

## Data Availability

All datasets generated for this study are included in the manuscript.

## Ethics Statement

Animals were maintained according to National Institute of Health (NIH) guidelines in AAALAC-accredited facilities. The animal facility was maintained at 21° ± 2°C, 50% ± 10% relative humidity and had a 12-h light:12-h dark cycle with lights on at 07:00 h (EST). The project protocol was approved under federal assurance (#D16-00028) by the Institutional Animal Care and Use Committee (IACUC) at the University of South Carolina.

## Author Contributions

RB and CM conceived and designed the experiments. LM, CM and RB performed the experiments. LM, KM and CM analyzed the data. LM, KM, RB and CM wrote the article and critical appraisal and approval of final manuscript.

## Conflict of Interest Statement

The authors declare that the research was conducted in the absence of any commercial or financial relationships that could be construed as a potential conflict of interest.
